# Differential toll-like receptor 3 (TLR3) expression and apoptotic response to TLR3 agonist in human neuroblastoma cells

**DOI:** 10.1186/1423-0127-18-65

**Published:** 2011-08-23

**Authors:** Jiin-Haur Chuang, Hui-Ching Chuang, Chao-Cheng Huang, Chia-Ling Wu, Yung-Ying Du, Mei-Lang Kung, Chih-Hao Chen, San-Cher Chen, Ming-Hong Tai

**Affiliations:** 1 Department of Surgery, Division of Pediatric Surgery, Chang Gung Memorial Hospital-Kaohsiung Medical Center, Kaohsiung, Taiwan; 2 Graduate Institute of Clinical Medical Sciences, Chang Gung University College of Medicine, Kaohsiung, Taiwan; 3 Department of Otolaryngology, Chang Gung Memorial Hospital-Kaohsiung Medical Center, Kaohsiung, Taiwan; 4 Department of Pathology, Chang Gung Memorial Hospital-Kaohsiung Medical Center, Kaohsiung, Taiwan; 5 Department of Biological Sciences, National Sun Yat-Sen University, Kaohsiung 804, Taiwan; 6 Division of Neurosurgery, Kaohsiung Veterans General Hospital, Kaohsiung 813, Taiwan; 7 Institute of Biomedical Sciences, National Sun Yat-Sen University, Kaohsiung 804, Taiwan

**Keywords:** neuroblastoma, toll-like receptor 3, poly(I:C), apoptosis

## Abstract

**Background:**

Toll-like receptor-3 (TLR-3) is a critical component of innate immune system against dsRNA viruses and is expressed in the central nervous system. However, it remains unknown whether TLR3 may serve as a therapeutic target in human neuroblastoma (NB).

**Methods:**

TLR3 expression in human NB samples was examined by immunohistochemical analysis. Quantitative RT-PCR and western blot was used to determine TLR3 expression in three human NB cell lines. The effect of TLR3 agonist, polyinosinic-polycytidylic acid (poly(I:C)), on the growth of human NB cells was evaluated by WST-1 cell proliferation assay, flow cytometry analysis, and immunoblot analysis. Blockade of TLR3 signaling was achieved using TLR3 neutralizing antibody, small interference RNA, and 2-aminopurine (2-AP), an inhibitor of protein kinase R (PKR), an interferon-induced, double-stranded RNA-activated protein kinase.

**Results:**

In immunohistochemical studies, TLR3 mainly expressed in the cytoplasm of ganglion cells and in some neuroblastic cells, but not in the stromal cells in human NB tissues. Among three human NB cell lines analyzed, TLR3 was significantly up-regulated in SK-N-AS cells at mRNA and protein level compared with other two low TLR3- expressing NB cells. Treatment with poly(I:C) elicited significant growth inhibition and apoptosis only in high TLR3-expressing SK-N-AS cells, but not in low TLR3-expressing SK-N-FI and SK-N-DZ cells. Moreover, poly(I:C) treatment significantly stimulated the activities of PKR, interferon regulatory factor 3 (IRF-3) and caspase-3 in SK-N-AS cells. Application of TLR3 neutralizing antibody or small interference RNA (siRNA) reduced the poly(I:C)-induced inhibition of cell proliferation and apoptosis in SK-N-AS cells. On the contrary, ectopic TLR3 expression enhanced the sensitivity of low TLR3-expressing NB cells to poly(I:C). Finally, application of 2-AP attenuated the poly(I:C)-induced IRF-3 and caspase-3 activation in SK-N-AS cells.

**Conclusion:**

The present study demonstrates that TLR3 is expressed in a subset of NB cells. Besides, TLR3/PKR/IRF-3/capase-3 pathway is implicated in the selective cytotoxicity of TLR3 agonist towards high TLR3-expressing NB cells.

## Introduction

Neuroblastoma (NB) accounts for more than 7% of malignancies in patients younger than 15 years old and 15% of all pediatric oncology deaths in the United States [1]. The disease is characterized by its broad range of clinical manifestations and versatile biological behaviors. Outcome of the patients with NB is poor for those with high-risk clinical phenotypes. Based on the latest report of the Italian Neuroblastoma Registry consisting of 781 NB children, the ten-year overall survival was 6.8% after progression and 14.4% after relapse [2]. A report from the Taiwan Children Cancer Foundation revealed that NB accounts for 6% of malignancies in children and the projected two-year survival rate was 59%, which dropped to 23% after disease progression [3].

The major factors influencing the survival of NB patients are age, stage and *MYCN *proto-oncogene status. Genomic *MYCN *amplification has been the most consistent genetic aberration associated with poor prognosis in NB [2,4-6]. Decrease of proliferation rate and induction of differentiation by a MYCN antisense DNA oligomer in human NB cell line may explain *MYCN *functions [7]. Over expression of *MYCN *transcriptional targets and low expression of neuronal differentiation genes predicts relapse and death from NB [8]. The MYCN oncoprotein was thus proposed as a drug development target [9]. However, recent evidence suggests that clinicopathological parameters, including tumor cell ploidy, localized disease and stage, may influence the prognosis of *MYCN *in NB [6], [10,11]. Two recent reports indicate that *MYCN *amplification alone is not sufficient to predict the outcome of the patients with NB [12,13]. Therefore, other factors affecting the response of NB cells to various stimuli may facilitate novel diagnostic or therapeutic targets for NB.

Toll-like receptors (TLRs) are human counterparts of Toll receptors in the fruit fly, *Drosophila*, which are originally implicated in the regulation of dorsoventral polarity, synaptogenesis, and path-finding in motor neuron growth cone [14]. There are 10 functional toll-like receptors (TLRs) in humans that specifically recognize pathogen-associated epitopes. While they are best known as initiators of the innate immune response to pathogens, TLRs are also expressed in glia and neurons of the central nervous system (CNS), which may recognize endogenous ligands and participate both in development and in responses associated with CNS injury [15]. Among the 10 functional TLRs, TLR2 are known to induce neural inflammation and neuronal damage, while TLR3 and TLR8 are negative regulators of axonal or neurite growth by inducing neuronal apoptosis [16-18].

The strategy of manipulating TLRs signaling is under active investigation for anti-neoplastic application. For example, stimulation of TLR9 with CpG oligonucleotide induces apoptosis of glioma and prolongs the survival of mice with experimental brain tumors [19]. TLR3 activation by its agonist directly triggers apoptosis in human breast cancer cells through activation of extrinsic caspases [20]. Moreover, a series of studies confirm the potential of TLR3 as therapeutic target for hepatoma, melanoma and clear cell renal carcinoma [21-24]. Synthetic agonists for several TLRs, including TLR3, TLR4, TLR7, TLR8, and TLR9, have been or are being developed for cancer treatment [25]. Despite of these studies, the role of TLR3 expression in NB remains largely unknown.

In the present study, we investigated TLR3 expression in human NB specimens to delineate the correlation of TLR3 expression with tumor differentiation. Subsequently, we analyzed TLR3 expression in three human NB cells and characterized two NB cells with differential TLR3 status. Finally, as TLR3 recognizes foreign double-stranded RNA (dsRNA), we treated these NB cells with TLR3 agonist, polyinosinic-polycytidylic acid (poly(I:C)), and monitored the difference in cellular proliferation, apoptosis, and expression profile of TLR3 signaling pathway.

## Materials and methods

### Immunohistochemical studies

Fourteen archival neuroblastic tumor specimens consisting of 8 cases of NB, 5 cases of ganglioneuroblastoma (GNB) and one ganglioneuroma (GN) were retrieved from the Department of Pathology, Chang Gung Memorial Hospital-Kaohsiung Medical Center (Kaohsiung, Taiwan). The use of archival tissues was approved by the institutional Internal Review Board of the hospital. In each case, well-preserved areas in paraffin-embedded NB tissues were reviewed and identified by a pathologist to prepare a tissues microarray of NB patients, which consisted of six to eight tissue cores (0.6 mm) from each patient.

Tissue specimens were maintained in formaldehyde-fixed, paraffin-embedded blocks. Sections stained with hematoxylin and eosin (H&E) were also reviewed. The paraffin sections from specimens were deparaffinized, blocked with 3% hydrogen peroxide for 10 min and subjected to antigen retrieval with microwave in 0.01 M citrate buffer for 7 min. The slides were then washed twice with PBS, incubated with TLR3 antibody (1:2000 dilution; Abcam, Cambridge, MA, USA) at room temperature for 30 min, followed by washing with TBST. Sections were detected with SuperPicTure Polymer detection kit (Zymed Laboratories, South San Francisco) for 30 min and developed with DAB chromogen (DAKO, USA) for 1 min. Sections were counterstained with Gill's hematoxylin, dehydrated and mounted with mounting medium.

The labeling index of TLR3 was calculated in percentage by two pathologists for each case. The immunoreactivity of TLR3 was graded according to the staining intensity as weak (1+), moderate (2+) and strong (3+) staining. Moderate or strong staining intensity was considered TLR3-positive. The percentages of positive cells were evaluated for neuroblastic cells and ganglion cells, respectively, in each neuroblastic tumor. Positive staining in more than 50% tumor cells was defined as "TLR3 overexpression" for either neuroblastic or ganglion cells. Negative was defined as <10% of area with staining.

### NB cell lines

Three human NB cell lines SK-N-AS, SK-N-FI and SK-N-DZ were purchased from American Type Culture Collection (Manassas, VA) and cultured with DMEM containing 10% (v/v) heat-inactivated fetal bovine serum (FBS; Invitrogen, Carlsbad, CA), L-glutamine, non-essential amino acids (Invitrogen; 10 mM) and antibiotic-antimycotic (Invitrogen) in a 5% CO2 humidified incubator at 37°C. The SK-N-AS cells were subcultured at 1:10 ratio when the cells grew to 80-90% of confluence, SK-N-DZ at 1:6 when 70-80% of confluence and SK-N-FI at 1:4 when 60% of confluence. To evaluate the effect of TLR3 agonist, the NB cells (seeded at 3 × 10^4 ^cells/cm^2 ^in 60-mm dishes) were treated with Poly(I:C) (Invivogen; San Diego, CA) in DMEM with 10% FBS at indicated doses for 24 h.

To verify the specificity of cellular response to poly(I:C), we also treated the NB cells with lipopolyssacharide (LPS) and CpG. Neuroblastoma cells were seeded in 60-mm culture dishes at a cell density of 3 × 10^4 ^cells/cm^2 ^and treated with 50 μg/ml Poly(I:C) (TLR3, Invivogen), 10 ng/ml LPS (TLR4, Sigma) or 1 μM CpG-ODN2006 (TLR9, InVivoGen) plus 10% FBS for 24 h.

### Quantitative RT-PCR (qRT-PCR) analysis

Total RNA was extracted by using TRIzol^® ^reagent (Invitrogen, Carlsbad, CA) from the three cell lines. After quantification, total RNA (2 μg) were used to produce cDNA by using the high capacity cDNA reverse transcription kit (ABI, Applied Biosystems, Foster City, CA) according to the manufacturer's recommendations. Expression levels of human TLR3 mRNA were determined by qRT-PCR using specific primers in PCR master mix (Yeastern Biotechnology; Taipei, Taiwan). The β-actin level was used as an internal control for normalization of TLR3 mRNA expression using the Prism 7700 Sequence Detection System (ABI). The primer sequences for qRT-PCR analysis of TLR3 and β-actin were: TLR3 forward 5'- TGGTTGGGCCACCTAGAAGT-3' and reverse, 5'- CCATTCCTGGCCTGTGAGTT -3'; β-actin forward 5'-TCACCCACACTGTGCCCATCTACGA-3' and 5'-CAGCGGAACCGCTCATTGCCAATGG-3'. The product size for TLR3 was 71 bp and for β-actin was 294 bp.

### Cell proliferation assay

Cells were seeded at a density of 5 × 10^3 ^cells/100 μL in 96-well plates and cultured overnight. The medium was changed, and the cells were then cultured in medium alone (control) or in medium containing different concentrations of poly(I:C). After treatment, 10 μL WST-1 reagent (Roche Diagnostics, Laval, Quebec, Canada) was added to each well and incubated for another 2 h at 37°C. The absorbance was determined using a microplate reader at a test wavelength of 450 nm and reference wavelength of 630 nm.

### Flow cytometry analysis

The percentage of cells in G0/G1, S, and G2/M phases was determined by flow cytometry analysis following propidium iodide (PI; Sigma, St. Louis, MS) staining. For PI staining, NB cells were seeded at a concentration of 1 × 10^6 ^cells/well in 3 ml medium containing 10% FBS in 6-well plates and left untreated or treated for 24 h or 48 h with poly(I:C). Cells were washed once with PBS and then fixed with ice-cold 70% PBS-ethanol for overnight at -20°C. Fixed cells were washed once with PBS and incubated in 1 ml PBS containing 10% Triton X-100, PI (20 mg/ml) and RNase A (Boehringer Mannheim, Indianapolis, IN) for 30 min at room temperature. Samples were analyzed using a FACS (BD Biosciences) and winMDI software.

### Western blot analysis

After treatment, cells were lysed with protein extraction solution containing proteases inhibitors (iNtRON Biotechnology) and the protein concentrations were measured with the BCA assay (Bio-Rad) with bovine serum albumin as standard. Thirty μg crude proteins were separated in 12-15% SDS-PAGE gels and transferred to nitrocellulose membrane. The membranes were immunoblotted overnight at 4°C with each primary antibody at indicated dilution. The primary antibodies included IRF-3 antibody (Epitomic, Inc.; Burlingame, CA), phosphor-IRF3 (pS386) antibody (Epitomic, Inc.), PKR antibody (Epitomic, Inc.), phosphor-PKR (pT451) antibody (Epitomic, Inc.), caspase-3 antibody (Cell Signaling Technology), and cleaved caspase-3 (Asp175) antibody (Cell Signaling Technology). Membranes were washed three times, and subjected to HRP-conjugated secondary antibody for 60 min at room temperature. Protein-antibody complexes were visualized with an ECL Western blotting detection and analysis system (Amersham Pharmacia Biotech, Uppsala, Sweden) and blots were exposed to film. Signals were quantified by densitometric analysis.

### TUNEL staining

TUNEL (terminal deoxynucleotidyl transferase-mediated deoxyuridine triphosphate (dUTP) nick end labeling) assay was used to detect fragmented DNA in SK-N-AS cells after Poly(I:C) treatment. Cells were grown in 12 well culture plate containing round glass cover slide. 24 h after treatment with Poly(I:C), the cells were fixed for 10 min at room temperature with 4% paraformaldehyde solution, washed three times with PBS. The TUNEL assay was performed according to the instruction manual of the In Situ Cell Death Detection Kit (Roche Diagnostics). Cells were incubated with TUNEL reaction mixture for 60 min at 37°C, protected from light, washed gently three times with PBS, and stained with DAPI for 5 min at room temperature in a dark chamber. After washing three times with PBS, cells were covered with cover slips. Photomicrographs were taken using a fluorescence Microscopy.

### TLR3 gene delivery

To increase the cellular TLR3 levels, SK-N-FI and SK-N-DZ NB cells were transfected with the mammalian expression vector encoding human TLR3 cDNA fused with hemagglutinin (HA) tag (OriGene Technologies Inc.; Rockville, MD) by Lipofectamine 2000 (Invitrogen; Carlsbad, CA).

### TLR3 RNA interference and antibodies neutralization

To knockdown endogenous TLR3 expression, human TLR3 stealth siRNA and control (scramble) siRNA were purchased from Invitrogen (Carlsbad, CA). The sequence for human TLR3 siRNA and control siRNA was 5'-CCTGAGCTGTCAAGCCACTACCTTT-3' and 5'-CCTGTCGAACTACCGCATCCAGTTT-3', respectively. Gene delivery of siRNA into NB cells was performed using Lipofectamine RNAiMAX (Invitrogen) following the manufacturer's protocol. After transfection, the cells were incubated with poly(I:C) 50 μg/ml for an additional 48 h before subsequent analysis.

For TLR3 antibody neutralization, NB cells (a density of 8 × 10^4 ^cells/ml in a 96-well plate) were incubated with TLR3 antibody (Santa Cruz Biotechnology; Santa Cruz, CA) at indicated concentrations for 1 h then treated with 50 μg/ml poly(I:C) for 24 h before subsequent analysis.

### Statistical analysis

All the data present in the figures were representation of at least triplicate experiments. Data were expressed as mean ± SD. Student's t-test was used for between-group comparison while analysis of variance was applied when more than 2 groups were compared. Statistical analysis of histological findings between different NB groups was performed using Fisher's extract test. A *P*-value less than 0.05 was considered statistically significant.

## Results

### TLR3 is expressed in the ganglion cells and differentiated neuroblastic cells in human neuroblastic tumors

**Figure 1:**
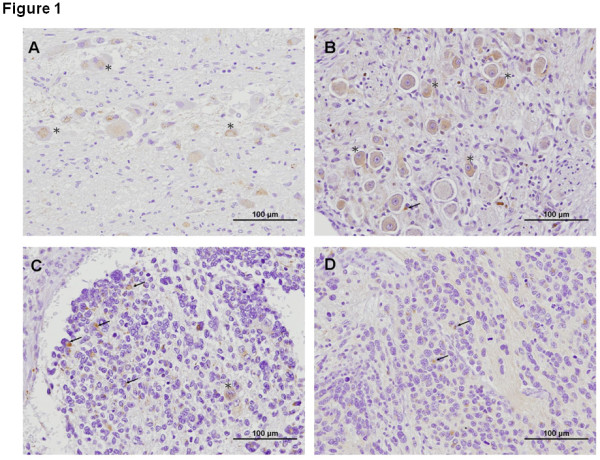
**TLR3 immunostaining in human neuroblastic tumors**. (A) TLR3 expression in ganglioneuroma (GN) and (B) in ganglioneuroblastoma (GNB). More than 50% of the ganglion cells (asterisk) and some neuroblastic cells (arrow) in GNB tissues exhibited prominent TLR3 staining. (C) TLR3 expression in differentiated NB tissues. Some neuroblastic (arrow) cells and sporadic ganglion cells (asterisk) exhibited TLR3 staining. (D) TLR3 expression in the undifferentiated NB tissues. Very few neuroblastic cells were positive for TLR3 expression. Original magnification, 200×.

To investigate TLR3 expression in human neuroblastic tumors, immunohistochemical analysis was performed using the archival specimens from 14 NB patients consisting of 8 cases of NB, 5 cases of ganglioneuroblastoma (GNB) and 1 case of ganglioneuroma (GN). Strong cytoplasmic TLR3 staining was observed in more than 50% of the ganglion cells in one GN and all GNB specimens (Figure 1A &1B). There was significant difference in TLR3 expression between GNB and NB tissues (Table 1). Interestingly, the TLR3-positive neuroblastic cells were mainly the differentiated NB cells with some characteristics of mature cells including nuclear enlargement, distinct cytoplasmic border, and cell processes, as well as presence of ganglion cells in sporadic sections (Figure 1C). However, TLR3 immunostaining was rarely detectable in the undifferentiated NB cells with characteristics of small, round, blue and dense nests of cells (Figure 1D). No TLR3 expression was detected in the stromal cells. The findings suggest the involvement of TLR3 in the tumorigenesis of NB and provide a rationale to target TLR3 with TLR3 agonists in vitro.

**Table 1:** Immunohisochemical analysis of TLR3 expression in human neuroblastoma tissues

	TLR 3 expression		
		
	Low expression^a^	High expression^b^	*P *value*
Neuroblastoma (n = 8)	8 (100%)	0	0.002
Ganglioneuroblastoma (n = 4)	0	4 (100%)	

^a^Low expression: the percentages of TLR3-positive cells less than 30% of total neuronal cells at low power field. ^b^High expression: the percentages of TLR3-positive cells ≧ 30% of total neuronal cells at low power field. *Analyzed with Fisher's exact probability test

### TLR3 is highly expressed in human SK-N-AS neuroblastic cells

**Figure 2:**
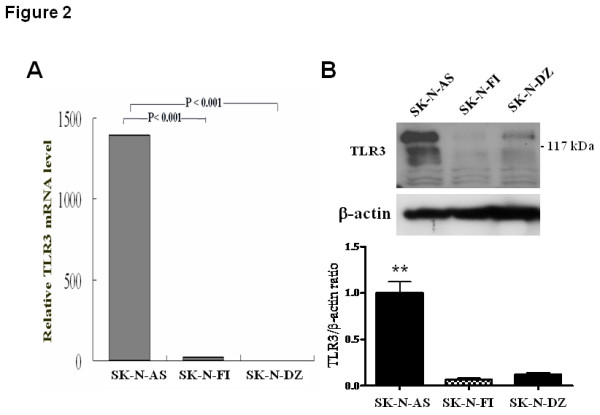
**TLR3 expression in human NB cell lines**. (A) Quantitative RT-PCR analysis of TLR3 mRNA level in three NB cell lines. (B) Western blot analysis of TLR3 protein level in three NB cell lines. Top panel, immunoblot analysis of TLR3 expression in three NB cell lines. Bottom panel, quantification results of TLR3 protein level were mean ± SD of triplicate experiments. **: *P *< 0.01.

Since TLR3 differentially expressed in human NB tissues, we examined TLR3 expression in the three human NB cell lines including SK-N-AS, SK-N-FI and SK-N-DZ cells. Quantitative RT-PCR showed that the TLR3 mRNA level was significantly higher in SK-N-AS cells than in the other NB cell lines (Figure 2A). Immunoblot analysis also revealed a higher TLR3 protein level in SK-N-AS cells than other two NB cells (Figure 2B). Thus, TLR3 is differentially expressed in human NB cell lines.

### TLR3 agonist preferentially induced growth inhibition and apoptosis in high TLR-3-expressing NB cells

**Figure 3:**
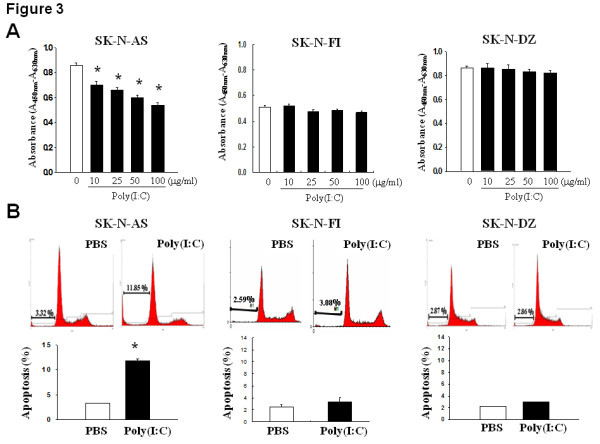
**Effect of TLR3 agonist on the proliferation and apoptosis of NB cell lines**. (A) Cell proliferation assay. After treatment with different doses of poly(I:C) for 24 h, the proliferation of various NB cells was measured by WST-1 assay and expressed as mean ± SD absorbance values of quadruplicate experiments. (B) Apoptosis assay. After treatment with 50 μg/ml poly(I:C) for 24 h, the apoptosis of various NB cells was determined by flow cytometry analysis following propidium iodide staining and expressed as mean ± SD percentages in pre-G_0 _stage from triplicate experiments. *: *P *< 0.05.

We subsequently examined whether the endogenous TLR3 status influenced the cellular response to TLR3 agonist, poly(I:C). By using WST-1 proliferation assay, it was observed that poly(I:C) treatment significantly inhibited the proliferation of high TLR3-expressing SK-N-AS cells in a dose-dependent manner (Figure 3A). In contrast, adding poly(I:C) had discernible effect on the proliferation of low TLR3-expressing SK-N-FI and SK-N-DZ cells.

We employed flow cytometry analysis to evaluate the apoptotic extent and cell cycle distribution in poly(I:C)-treated NB cells. It was found that poly(I:C) treatment significantly increased the percentages of cells in pre-G0 phase in SK-N-AS cells after treatment for 24 hours (Figure 3B). The poly(I:C)-induced apoptosis of SK-N-AS cells was also validated by TUNEL assay (Additional file 1, figure S1). However, poly(I:C) treatment had no significant effect on cell proliferation or apoptosis in SK-N-FI and SK-N-DZ cells even after extended exposure for 48 hours (Figure 3B). Thus, TLR3 agonist preferentially inhibited the growth of high TLR3-expressing NB cells.

### TLR3 agonist stimulated the PKR/IRF-3/caspase-3 pathway in high TLR3-expressing NB cells

**Figure 4:**
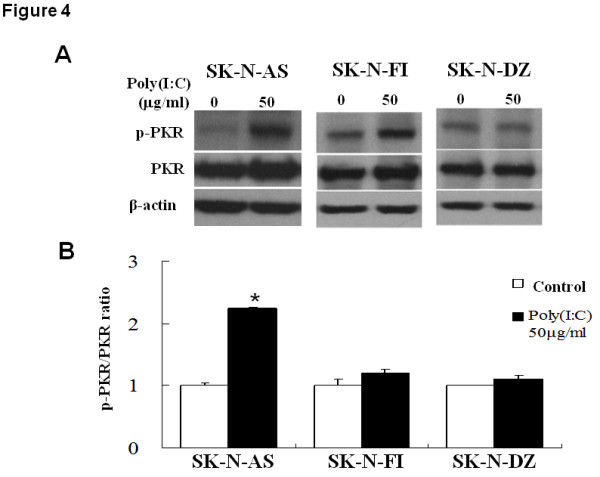
**Effect of TLR3 agonist on PKR expression and phosphorylation in NB cell lines**. (A) Western blot analysis. After treatment with 50 μg/ml poly(I:C) for 24 h, NB cells were harvested and subjected to immunoblot analysis. (B) Quantification of PKR activation in NB cells after poly(I:C) treatment. The ratio of phosphorylated PKR level over total PKR level was determined and expressed as mean ± SD from triplicate experiments. *: *P *< 0.05.

Since PKR is essential for dsRNA signaling, we evaluated whether poly(I:C) also caused changes in PKR protein level and phosphorylation in the three NB cells. It was shown that poly(I:C) treatment significantly enhanced the phosphorylation of PKR in SK-N-AS cells by more than 2-fold of control (Figure 4). On the contrary, neither PKR expression nor phosphorylation in SK-N-FI and SK-N-DZ cells was affected by TLR3 agonist. Thus, TLR3 agonist induced activation of PKR signaling in NB cells with high TLR3 levels.

Interferon regulatory factor 3 (IRF-3) is known to undergo phosphorylation-induced activation in virus-infected cells and plays an important role in the antiviral innate immune response under the regulation of PKR [26]. It was found that application of poly(I:C) significantly elevated the phosphorylation of IRF-3 in SK-N-AS cells (data not shown). Interestingly, poly(I:C) treatment also elicited a moderate, yet significant elevation of phosphorylated IRF-3 in SK-N-FI cells, but not in SK-N-DZ cells.

**Figure 5:**
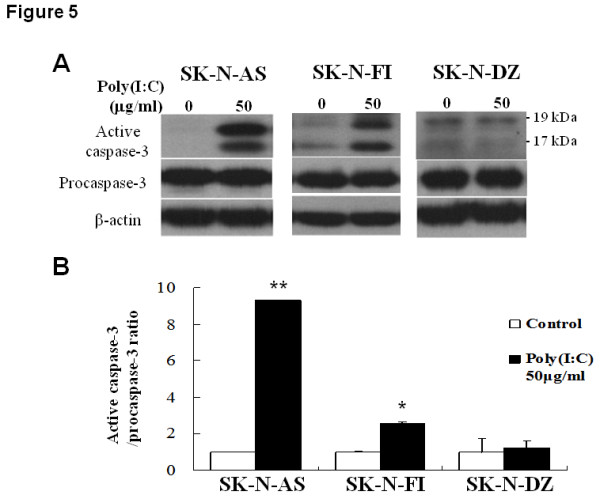
**Effect of TLR3 agonist on caspase-3 activation in NB cell lines**. (A) Western blot analysis. After treatment with 50 μg/ml poly(I:C) for 24 h, NB cells were harvested and subjected to immunoblot analysis. (B) Quantification of active caspase-3 in NB cells after poly(I:C) treatment. The ratio of active caspase-3 over procaspase-3 level was determined and expressed as mean ± SD from triplicate experiments. **: *P *< 0.01. *: *P *< 0.05.

Since dsRNA induces cell death through activation of caspases, we measured the level of procaspase-3 and active caspase-3 in NB cells after poly(I:C) treatment. There was no significant change of procaspase-3 level in the three NB cells at any time point after poly(I:C)treatment. However, active caspase-3 was significantly up-regulated in SK-N-AS cells at after treatment for 24 hours (Figure 5). A similar trend was also found in SK-N-FI, in which poly(I:C) treatment induced milder, yet significant elevation of active caspase-3. Again, active caspase-3 was not found in poly(I:C)-treated SK-N-DZ cells. Therefore, TLR3 agonist induced apoptosis in high TLR3-expressing SK-N-AS cells via caspase-3 activation.

To verify the specificity of cellular response to poly(I:C), NB cells were also challenged with other TLR agonists, including LPS and CpG, then examined for capsase-3 activation. However, LPS and CpG treatment failed to elicit caspase-3 activation in all the three NB cells (Additional file 2, figure S2)

### Blockade of TLR3 signaling using neutralizing antibody or small interference RNA (siRNA) attenuated the poly(I:C)-induced growth inhibition and apoptosis in SK-N-AS cells

**Figure 6:**
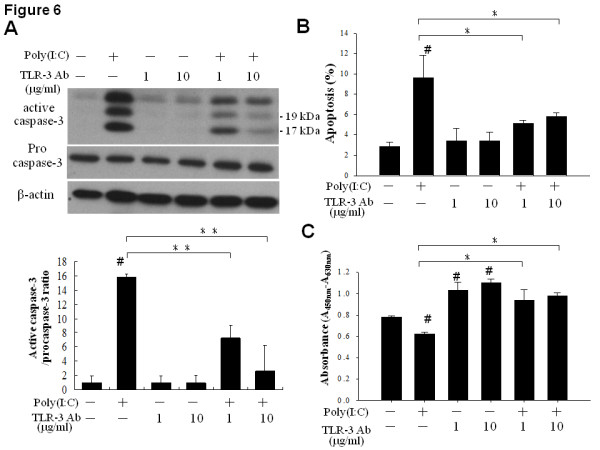
**Effect of TLR3 neutralization on poly(I:C)-induced apoptosis in SK-N-AS cells**. (A) Effect of TLR3 neutralization on poly(I:C)-induced caspase-3 activation. After incubation with TLR3 antibodies of indicated doses for 1 h, SK-N-AS cells were treated with poly(I:C) (50 μg/ml) for 24 h, and then harvested for immunoblot analysis (top panel). Arrows indicated the molecular weight of active caspase-3 at 17 kDa and 19 kDa, respectively. Quantification results of caspase-3 activation in different treatment groups were expressed as mean ± SD from triplicate experiments (bottom panel). #: *P *< 0.05 versus non-treated control, **: *P *< 0.01. (B) Flow cytometry analysis of poly(I:C)-induced apoptosis in SK-N-AS cells after TLR3 neutralization. Data were mean ± SD from triplicate experiments. #: *P *< 0.05 versus non-control, *: *P *< 0.05. (C) Cell proliferation assay of poly(I:C)-induced growth inhibition in SK-N-AS cells after TLR3 neutralization. Data were mean ± SD from quadruplicate experiments. #: *P *< 0.05 versus non-control, *: *P *< 0.05.

To confirm TLR3 is directly involved in the poly(I:C)-induced apoptosis, we employed TLR3 neutralizing antibodies to disrupt the TLR3 signaling in SK-N-AS cells. It was found that caspase-3 activation was significantly perturbed by TLR3 antibodies blockade (Figure 6A), but not by non-specific antibody (Additional file 3, figure S3). Flow cytometry analysis further revealed that TLR3 antibody neutralization significantly reversed the poly(I:C)-induced apoptosis in SK-N-AS cells (Figure 6B). By cell proliferation assay, it was found that prior incubation with TLR3 antibody significantly increased the proliferation of SK-N-AS cells, thereby perturbing the growth inhibition mediated by poly(I:C) treatment (Figure 6C).

**Figure 7:**
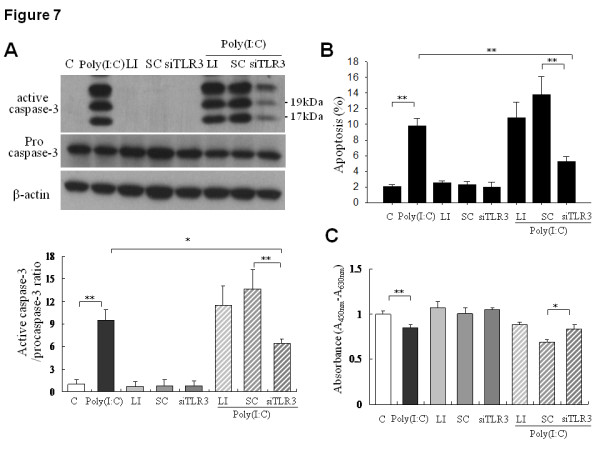
**Effect of TLR3 knock-down on poly(I:C)-induced apoptosis in SK-N-AS cells**. After transfection with siRNA (60 nM) for 24 h, the cells were incubated with poly(I:C) 50 μg/ml or vehicle for another 24 h and were then harvested for assay. C, control; LI, lipofectamine; SC. Scramble control siRNA; siTLR3, TLR3 siRNA. (A) Effect of TLR3 siRNA knockdown on poly(I:C)-induced caspase-3 activation. Arrows indicated the molecular weight of active caspase-3 at 17 and 19 kDa, respectively. Quantification results of caspase-3 activation in different treatment groups were expressed as mean ± SD from triplicate experiments (bottom panel). *: *P *< 0.05, **: *P *< 0.01. (B) Flow cytometry analysis of poly(I:C)-induced apoptosis in SK-N-AS cells after TLR3 siRNA knockdown. Data were mean ± SD from triplicate experiments. **: *P *< 0.01. (C) Cell proliferation assay of poly(I:C)-induced growth inhibition in SK-N-AS cells after siRNA knock-down of TLR3. Data were mean ± SD from triplicate experiments. *: *P *< 0.05.

To further validate the role of TLR3 in poly(I:C)-induced apoptosis, SK-N-AS cells were transfected with TLR3-specific small interference RNA (TLR3 siRNA) then monitored for cellular responses to poly(I:C). It was found that gene delivery of TLR3 siRNA, but not control siRNA, led to significant reduction (more than 50%) of TLR3 mRNA level (Additional file 4, figure S4). Besides, TLR3 knockdown significantly reversed the poly(I:C)-induced caspase-3 activation, apoptosis and growth inhibition in SK-N-AS cells (Figure 7). Together, these results supported the pro-apoptotic and anti-proliferative function of TLR3 signaling in human NB cells.

### Ectopic TLR3 expression rendered the low TLR3-expressing NB cells sensitive to poly(I:C) treatment

**Figure 8:**
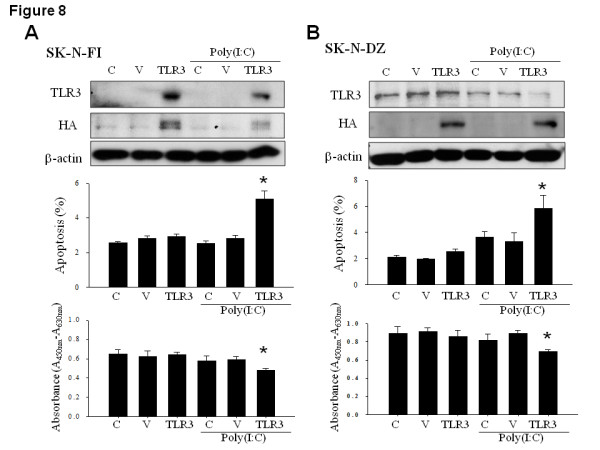
**Effect of ectopic TLR3 overexpression on the poly(I:C) sensitivity in low TLR3-expressing NB cells**. (A, B top panel) Immunoblot analysis of endogenous and exogenous TLR3 expression in transfected SK-N-FI and SK-N-DZ cells in the absence or presence of a 24-h poly(I:C) treatment. C: control, V: vector, TLR3: TLR3-expressing vector. (A, B middle panel) Flow cytometry analysis of apoptotic response in TLR3-transduced SK-N-FI and SK-N-DZ cells in the absence or presence of poly(I:C) treatment. Data were mean ± SD from triplicate experiments. *: *P *< 0.05. (A, B bottom panel) Cell proliferation assay of TLR3-transduced SK-N-FI and SK-N-DZ cells in the absence or presence of poly(I:C) treatment. Data were mean ± SD from quadruplicate experiments *: *P *< 0.05.

Subsequently, we examined the feasibility of sensitizing the low TLR3-expressing NB cells to poly(I:C) by ectopic TLR3 overexpression. After transfection with TLR-3-expressing vector, it was observed that TLR3 gene delivery significantly elevated the HA-positive, exogenous TLR3 level in SK-N-FI and SK-N-DZ cells (Figure 8A). Despite the lack of effect on apoptosis and cell proliferation by itself, transient TLR3 expression significantly augmented the poly(I:C)-mediated apoptosis and growth inhibition in both low TLR3-expressing cells. This also seemed in accordance with the reduction of TLR3 transgene level in NB cells upon simultaneous poly(I:C) application. Thus, ectopic TLR3 expression indeed sensitized the low TLR3-expressing NB cells to poly(I:C) treatment.

### Pharmaceutical inhibition of dsRNA-regulated protein kinase (PKR) attenuated poly(I:C)-induced action of PKR/IRF3/caspase 3 pathway

To determine the role of dsRNA-regulated protein kinase (PKR) in TLR3-mediated cell death, SK-N-AS cells were treated with PKR inhibitor, 2-aminopurine (2-AP), followed by poly(I:C) administration. 2-AP significantly blocked the expression of p-PKR (Additional file 5, figure S5), reduced the expression of p-IRF3 (Additional file 5, figure S5B) from 3 h up till 24 h after treatment, resulting in significant reduction of activated caspase-3 expression at 24 h in SK-N-AS (Additional file 5, figure S5C).

## Discussion

The first report that TLR3 can directly trigger apoptosis in human breast cancer cells opened a new avenue for cancer therapeutics [20]. The observation quickly gained support from other studies that confirm a role for TLR3 in the tumorigenesis of hepatoma, melanoma and clear cell renal carcinoma [21-24]. In this study, we provided the first evidence that TLR3 is differentially expressed in human NB tissues and cells. The histological study in human NB tissues reveals that TLR3 expression is present mainly in the cytoplasm of ganglion cells of GNB, but rare or not detectable in the neuroblastic cells of NB. Besides, TLR3 is not present in the stromal cells. These findings indicate heterogeneous neuroblastic cell types in human neuroblastic tumors that harbor differential TLR3 levels and may respond differently to TLR3 agonist.

Histological studies revealed that TLR3 expresses mainly in the cytoplasm of ganglion cells in GNB tissues. In contrast, TLR3 was rarely detected in neuroblastic cells of NB tissues. There is an excellent correlation between differentiation and apoptosis in NB tissues. Hoehner *et al*. found that the most differentiated NB cells lie adjacent to TUNEL-positive, morphologically apoptotic cells [27]. Another study also showed that apoptosis in neuroblastic tumor is present mainly in cases with well differentiation status and favorable outcome [27]. The above findings implicate that the interplay between differentiation and apoptosis may be involved in the regression of neuroblastic tumors [27]. Our findings of TLR3 expression in the more differentiated NB tissues and TLR3 signaling during apoptosis of NB cell lines seem consistent with the above notions. However, due to the relative small sample size in this study, future studies are warranted to validate the role of TLR3 signaling in differentiation and apoptosis in NB.

Poly(I:C) is known to transmit anti-viral and inflammatory signaling in TLR3-expressing astrocytes through activation of PKR [28]. PKR is also the essential component of TLR3-mediated NF-_κ_B activation in human epidermal keratinocytes [29]. Another study also reported that PKR was required for maximal type I IFN-beta induction and the induction of apoptosis in different cell lines by both transfected T7 phage polymerase-synthesized RNA and Poly(I:C) [30]. In cultured human biliary epithelial cells, stimulation with poly(I:C) induces the activation of both transcription factors NF-_κ_B and IRF3 and the production of interferon-beta1 (IFN-beta1) and MxA as potent antiviral responses [31]. The above studies are consistent with our studies in NB cells, indicating that TLR3/PKR/IRF3 signaling components are not only for innate immune response against viral infection, and probably respond to tumor antigens, in various human cell lines including NB cells.

It remains elusive how TLR3 executes its function upon ligand stimulation in NB cells. TLR3 is known to be localized in the endoplasmic reticulum (ER) of unstimulated cells [32,33]. In response to ligand stimulation, TLR3 may move to endosomes or other compartments to execute antiviral activities or inflammatory cytokine production [34]. Two ER proteins, glucose-regulated protein 78 (GRP78) and calreticulin, have been identified as independent prognostic factors for NB patients [35,36]. Our pilot study revealed that poly(I:C) induced significant upregulation of GRP78 and calreticulin in SK-N-AS cells, but not in SK-N-FI and SK-N-DZ (Chuang *et al*.; unpublished observation). Thus, TLR3 signaling may affect the expression of some ER proteins and control the cell fate or differentiation of high TLR3-expressing NB cells. An interesting observation reveals the association of TLRs with other ER membrane proteins such as UNC93B. Single point mutation (H412R) in UNC93B abolishes signaling via TLR3, 7, and 9 [37]. The latter suggests a physical association between UNC93B and TLRs in the ER is probably essential for proper TLR signaling in high TLR3-expressing NB cells.

**Figure 9:**
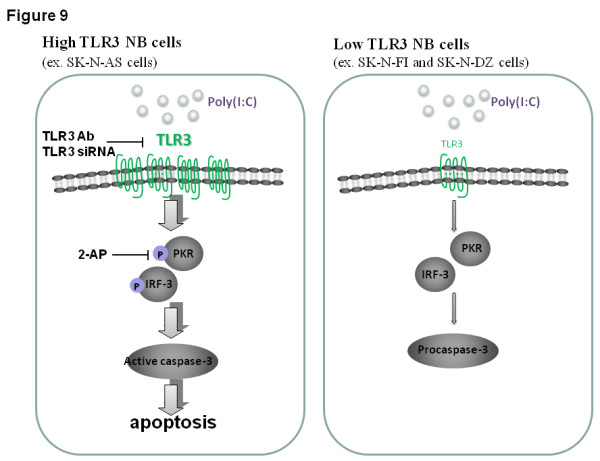
**Scheme for poly(I:C)-induced apoptotic signaling pathway in high TLR3-expressing NB cells (left panel), but not low TLR3-expressing NB cells (right panel)**.

In this study, both TLR3 antibody and siRNA can attenuate the poly(I:C)-induced inhibition of cell proliferation as well as apoptosis, which was most prominent in SK-N-AS cells (Figure 9). One interesting finding is that treatment of NB cells with either TLR3 antibody or TLR3 siRNA is able to increase cell proliferation in all the three NB cells. The findings are consistent with previous reports, which reveal that TLR3 signaling is a negative regulator of embryonic neural progenitor cell proliferation or liver regeneration (46, 47). Blockade of TLR3 signaling may therefore reverse the function and augment NB cell proliferation.

In summary, the present study unveiled the differential TLR3 expression in different types of NB specimens. Moreover, this study demonstrated the susceptibility of high TLR3-expressing NB cells to agonist-induced apoptosis and the involvement of PKR/IRF3/caspase 3 signaling pathways. Further studies with the aid of pretreatment with interferon alpha [38], simultaneous administration of CpG DNA [19,39], or targeting MYCN or MDA5 may be helpful in augmenting the poly(I:C) sensitivity and NB immunotherapy. As an intracytoplasmic RNA sensor, MDA5 expression was higher in SK-N-FI (unpublished data), which also responded to poly(I:C) treatment and might explain why active caspase-3 expression was higher in SK-N-FI than in SK-N-DZ, despite both were lower than that in SK-N-AS.

## Conclusion

This study unveiled that TLR3 is expressed in a subset of relatively benign NB cells. Besides, TLR agonist induced proapoptotic activity in NB cells with high endogenous TLR3 level. Genetic manipulation data further support that the cellular TLR3 level determined the sensitivity of NB cells to poly(I:C)-induced cell death. Our study may open a new avenue of therapeutic indication for TLR3 agonists as an adjuvant therapy in intractable NB.

## List of Abbreviations

(dsRNA): Double-stranded RNA; (GNB): Ganglioneuroblastoma; (IRF3): Interferon regulatory factor 3; (NB): Neuroblastoma; (PKR): Double-stranded RNA-dependent protein kinase; (poly(I:C): Polyinosinic-polycytidylic acid); (TLR): Toll-like receptor.

## Competing interests

The authors declare that they have no competing interests.

## Authors' contributions

JCC conceived of the study, coordinated all studies and drafted the manuscript; HCC carried out the apoptosis studies in NB cell lines; CCH analyzed TLR3 expression in NB samples; CLW participated in flow cytometry analysis; YYD performed immunoblot analysis and caspase-3 assay; MLK performed TLR3 overexpression and knockdown experiments; CHC collected and archived NB samples; SCC assisted the manuscript preparation and proofreading; MHT conceived of the study, designed the experiments, and accomplished manuscript preparation. All authors read and approved the final manuscript.

## Supplementary Material

Additional file 1 **Figure S1**. TUNEL (terminal deoxynucleotidyl transferase-mediated deoxyuridine triphosphate (dUTP) nick end labeling) assay was used to detect fragmented DNA in SK-N-AS cells 24 h after poly(I:C) treatment. DAPI was used for staining of the nucleus. Positive immunostaing was present only in some NB cells after Poly(I:C) treatment, but not in control. Click here for file

Additional file 2 **Figure S2**. Effect of TLR agonists on caspase-3 activation in NB cells. NB cells were treated with TLR agonists including TLR3 agonist (Poly(I:C); 50 μg/ml), TLR4 agonist (LPS; 10 ng/ml), and TLR9 agonist (CpG-ODN2006; 1 μM) for 24 h and subjected to immunoblot analysis of caspase-3 activation. Click here for file

Additional file 3 **Figure S3**. Neutralization of TLR3 in SK-N-AS revealed that TLR3 specific antibody, in comparison with non-specific IgG control, down-regulated the expression of active caspase-3 expression. However, for uncertain reason, high dose of IgG may also exert some neutralization effect. Click here for file

Additional file 4 **Figure S4**. Transfection of siRNA targeting TLR3 or scramble RNA was evaluated by using QRT-PCR to detect TLR3 mRNA. TLR3 mRNA expression decreased to 43% in SK-N-AS cells treated with siRNA targeting TLR3 (siTLR3), but not in scramble or lipofectamine control. Click here for file

Additional file 5 Figure S5. Effect of PKR inhibitor, 2-aminopurine (2-AP), on poly(I:C)-induced suppression of PKR and IRF3 expression and caspase-3 activation in SK-N-AS cells. (A) NB cells were pretreated with 2-AP (10 mM) for 5 min, followed by treatment with 50 μg/ml poly(I:C) for 24 h, then harvested for immunoblot analysis. (A) Immunoblot analysis of total and phosphorylated PKR (top panel). Quantification of PKR activation was determined using phosphorylated PKR/total PKR (bottom panel). Data were mean ± SD from triplicate experiments. *: *P *< 0.05. **: *P *< 0.01. (B) Immunoblot analysis of total and phosphorylated IRF-3 (top panel). Quantification of IRF3 activation was determined using phosphorylated IRF3/total IRF3 (bottom panel). Data were mean ± SD from triplicate experiments. **: *P *< 0.01. (C) Immunoblot analysis of pro- and activated caspase-3 (top panel). Quantification of caspase 3 activation was determined using activated caspase-3 level over procaspase-3 level (bottom panel). Data were mean ± SD from triplicate experiments. **: *P *< 0.01. Click here for file

## References

[B1] Maris JM Hogarty MD Bagatell R Cohn SL Neuroblastoma Lancet 2007 369 9579 2106 2120 10.1016/S0140-6736(07)60983-0 17586306

[B2] Garaventa A Parodi S De Bernardi B Dau D Manzitti C Conte M Casale F Viscardi E Bianchi M D'Angelo P Outcome of children with neuroblastoma after progression or relapse. A retrospective study of the Italian neuroblastoma registry Eur J Cancer 2009 45 16 2835 2842 10.1016/j.ejca.2009.06.010 19616426

[B3] Taiwan Pediatric Oncology Group N2002 Follow-Up Report

[B4] Brodeur GM Seeger RC Schwab M Varmus HE Bishop JM Amplification of N-myc in untreated human neuroblastomas correlates with advanced disease stage Science 1984 224 4653 1121 1124 10.1126/science.6719137 6719137

[B5] Seeger RC Brodeur GM Sather H Dalton A Siegel SE Wong KY Hammond D Association of multiple copies of the N-myc oncogene with rapid progression of neuroblastomas N Engl J Med 1985 313 18 1111 1116 10.1056/NEJM198510313131802 4047115

[B6] Schneiderman J London WB Brodeur GM Castleberry RP Look AT Cohn SL Clinical significance of MYCN amplification and ploidy in favorable-stage neuroblastoma: a report from the Children's Oncology Group J Clin Oncol 2008 26 6 913 918 10.1200/JCO.2007.13.9493 18281664

[B7] Negroni A Scarpa S Romeo A Ferrari S Modesti A Raschella G Decrease of proliferation rate and induction of differentiation by a MYCN antisense DNA oligomer in a human neuroblastoma cell line Cell Growth Differ 1991 2 10 511 518 1751406

[B8] Fredlund E Ringner M Maris JM Pahlman S High Myc pathway activity and low stage of neuronal differentiation associate with poor outcome in neuroblastoma Proc Natl Acad Sci USA 2008 105 37 14094 14099 10.1073/pnas.0804455105 18780787 PMC2544584

[B9] Lu X Pearson A Lunec J The MYCN oncoprotein as a drug development target Cancer Lett 2003 197 1-2 125 130 10.1016/S0304-3835(03)00096-X 12880971

[B10] Cohn SL Look AT Joshi VV Holbrook T Salwen H Chagnovich D Chesler L Rowe ST Valentine MB Komuro H Lack of correlation of N-myc gene amplification with prognosis in localized neuroblastoma: a Pediatric Oncology Group study Cancer Res 1995 55 4 721 726 7850780

[B11] Perez CA Matthay KK Atkinson JB Seeger RC Shimada H Haase GM Stram DO Gerbing RB Lukens JN Biologic variables in the outcome of stages I and II neuroblastoma treated with surgery as primary therapy: a children's cancer group study J Clin Oncol 2000 18 1 18 26 10623689 10.1200/JCO.2000.18.1.18

[B12] Bagatell R Rumcheva P London WB Cohn SL Look AT Brodeur GM Frantz C Joshi V Thorner P Rao PV Outcomes of children with intermediate-risk neuroblastoma after treatment stratified by MYCN status and tumor cell ploidy J Clin Oncol 2005 23 34 8819 8827 10.1200/JCO.2004.00.2931 16314642

[B13] Suita S Tajiri T Kaneko M Hirai M Mugishima H Sugimoto T Tsuchida Y Implications of MYCN amplification in patients with stage 4 neuroblastoma who undergo intensive chemotherapy J Pediatr Surg 2007 42 3 489 493 10.1016/j.jpedsurg.2006.10.056 17336185

[B14] Rose D Zhu X Kose H Hoang B Cho J Chiba A Toll, a muscle cell surface molecule, locally inhibits synaptic initiation of the RP3 motoneuron growth cone in Drosophila Development 1997 124 8 1561 1571 9108372 10.1242/dev.124.8.1561

[B15] Larsen PH Holm TH Owens T Toll-like receptors in brain development and homeostasis Sci STKE 2007 2007 402 pe47. 17785714 10.1126/stke.4022007pe47

[B16] Cameron JS Alexopoulou L Sloane JA DiBernardo AB Ma Y Kosaras B Flavell R Strittmatter SM Volpe J Sidman R Toll-like receptor 3 is a potent negative regulator of axonal growth in mammals J Neurosci 2007 27 47 13033 13041 10.1523/JNEUROSCI.4290-06.2007 18032677 PMC4313565

[B17] Hoffmann O Braun JS Becker D Halle A Freyer D Dagand E Lehnardt S Weber JR TLR2 mediates neuroinflammation and neuronal damage J Immunol 2007 178 10 6476 6481 17475877 10.4049/jimmunol.178.10.6476

[B18] Ma Y Li J Chiu I Wang Y Sloane JA Lu J Kosaras B Sidman RL Volpe JJ Vartanian T Toll-like receptor 8 functions as a negative regulator of neurite outgrowth and inducer of neuronal apoptosis J Cell Biol 2006 175 2 209 215 10.1083/jcb.200606016 17060494 PMC2064562

[B19] El Andaloussi A Sonabend AM Han Y Lesniak MS Stimulation of TLR9 with CpG ODN enhances apoptosis of glioma and prolongs the survival of mice with experimental brain tumors Glia 2006 54 6 526 535 10.1002/glia.20401 16906541

[B20] Salaun B Coste I Rissoan MC Lebecque SJ Renno T TLR3 can directly trigger apoptosis in human cancer cells J Immunol 2006 176 8 4894 4901 16585585 10.4049/jimmunol.176.8.4894

[B21] Khvalevsky E Rivkin L Rachmilewitz J Galun E Giladi H TLR3 signaling in a hepatoma cell line is skewed towards apoptosis J Cell Biochem 2007 100 5 1301 1312 10.1002/jcb.21119 17243100

[B22] Morikawa T Sugiyama A Kume H Ota S Kashima T Tomita K Kitamura T Kodama T Fukayama M Aburatani H Identification of Toll-like receptor 3 as a potential therapeutic target in clear cell renal cell carcinoma Clin Cancer Res 2007 13 19 5703 5709 10.1158/1078-0432.CCR-07-0603 17908959

[B23] Salaun B Lebecque S Matikainen S Rimoldi D Romero P Toll-like receptor 3 expressed by melanoma cells as a target for therapy? Clin Cancer Res 2007 13 15 Pt 1 4565 4574 17671143 10.1158/1078-0432.CCR-07-0274

[B24] Jiang Q Wei H Tian Z Poly I:C enhances cycloheximide-induced apoptosis of tumor cells through TLR3 pathway BMC Cancer 2008 8 12 10.1186/1471-2407-8-12 18199340 PMC2242792

[B25] Krieg AM Development of TLR9 agonists for cancer therapy J Clin Invest 2007 117 5 1184 1194 10.1172/JCI31414 17476348 PMC1857270

[B26] Zhang P Samuel CE Induction of protein kinase PKR-dependent activation of interferon regulatory factor 3 by vaccinia virus occurs through adapter IPS-1 signaling J Biol Chem 2008 283 50 34580 34587 10.1074/jbc.M807029200 18927075 PMC2596378

[B27] Mejia C Navarro S Llombart-Bosch A Apoptosis in peripheral neuroblastic tumors. Immunohistochemical expression of bcl-2 and p53 is related to DNA fragmentation Histol Histopathol 2007 22 12 1365 1370 17701916 10.14670/HH-22.1365

[B28] Scumpia PO Kelly KM Reeves WH Stevens BR Double-stranded RNA signals antiviral and inflammatory programs and dysfunctional glutamate transport in TLR3-expressing astrocytes Glia 2005 52 2 153 162 10.1002/glia.20234 15920723

[B29] Kalali BN Kollisch G Mages J Muller T Bauer S Wagner H Ring J Lang R Mempel M Ollert M Double-stranded RNA induces an antiviral defense status in epidermal keratinocytes through TLR3-, PKR-, and MDA5/RIG-I-mediated differential signaling J Immunol 2008 181 4 2694 2704 18684960 10.4049/jimmunol.181.4.2694

[B30] McAllister CS Samuel CE The RNA-activated protein kinase enhances the induction of interferon-beta and apoptosis mediated by cytoplasmic RNA sensors J Biol Chem 2009 284 3 1644 1651 19028691 10.1074/jbc.M807888200 PMC2615529

[B31] Harada K Sato Y Itatsu K Isse K Ikeda H Yasoshima M Zen Y Matsui A Nakanuma Y Innate immune response to double-stranded RNA in biliary epithelial cells is associated with the pathogenesis of biliary atresia Hepatology 2007 46 4 1146 1154 10.1002/hep.21797 17661372

[B32] Johnsen IB Nguyen TT Ringdal M Tryggestad AM Bakke O Lien E Espevik T Anthonsen MW Toll-like receptor 3 associates with c-Src tyrosine kinase on endosomes to initiate antiviral signaling EMBO J 2006 25 14 3335 3346 10.1038/sj.emboj.7601222 16858407 PMC1523188

[B33] Latz E Schoenemeyer A Visintin A Fitzgerald KA Monks BG Knetter CF Lien E Nilsen NJ Espevik T Golenbock DT TLR9 signals after translocating from the ER to CpG DNA in the lysosome Nat Immunol 2004 5 2 190 198 10.1038/ni1028 14716310

[B34] Ivanov S Dragoi AM Wang X Dallacosta C Louten J Musco G Sitia G Yap GS Wan Y Biron CA A novel role for HMGB1 in TLR9-mediated inflammatory responses to CpG-DNA Blood 2007 110 6 1970 1981 10.1182/blood-2006-09-044776 17548579 PMC1976374

[B35] Hsu WM Hsieh FJ Jeng YM Kuo ML Chen CN Lai DM Hsieh LJ Wang BT Tsao PN Lee H Calreticulin expression in neuroblastoma--a novel independent prognostic factor Ann Oncol 2005 16 2 314 321 10.1093/annonc/mdi062 15668290

[B36] Hsu WM Hsieh FJ Jeng YM Kuo ML Tsao PN Lee H Lin MT Lai HS Chen CN Lai DM GRP78 expression correlates with histologic differentiation and favorable prognosis in neuroblastic tumors Int J Cancer 2005 113 6 920 927 10.1002/ijc.20693 15514946

[B37] Brinkmann MM Spooner E Hoebe K Beutler B Ploegh HL Kim YM The interaction between the ER membrane protein UNC93B and TLR3, 7, and 9 is crucial for TLR signaling J Cell Biol 2007 177 2 265 275 10.1083/jcb.200612056 17452530 PMC2064135

[B38] Kaiser WJ Kaufman JL Offermann MK IFN-alpha sensitizes human umbilical vein endothelial cells to apoptosis induced by double-stranded RNA J Immunol 2004 172 3 1699 1710 14734752 10.4049/jimmunol.172.3.1699

[B39] Whitmore MM DeVeer MJ Edling A Oates RK Simons B Lindner D Williams BR Synergistic activation of innate immunity by double-stranded RNA and CpG DNA promotes enhanced antitumor activity Cancer Res 2004 64 16 5850 5860 10.1158/0008-5472.CAN-04-0063 15313929

