# Supplementary figures and images for: Differential toll-like receptor 3 (TLR3) expression and apoptotic response to TLR3 agonist in human neuroblastoma cells
Source: J Biomed Sci. 2011 Aug 23;18(1):65. doi: 10.1186/1423-0127-18-65 (PMC3184062; doi:10.1186/1423-0127-18-65)

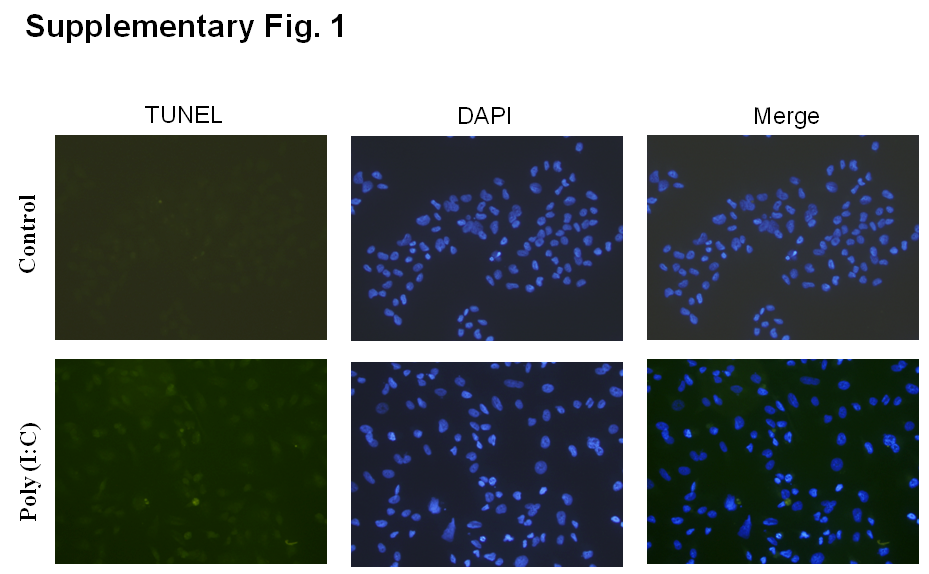

Supplement: Additional file 1 — Figure S1. TUNEL (terminal deoxynucleotidyl transferase-mediated deoxyuridine triphosphate (dUTP) nick end labeling) assay was used to detect fragmented DNA in SK-N-AS cells 24 h after poly(I:C) treatment. DAPI was used for staining of the nucleus. Positive immunostaing was present only in some NB cells after Poly(I:C) treatment, but not in control. [file 1423-0127-18-65-S1.TIFF]

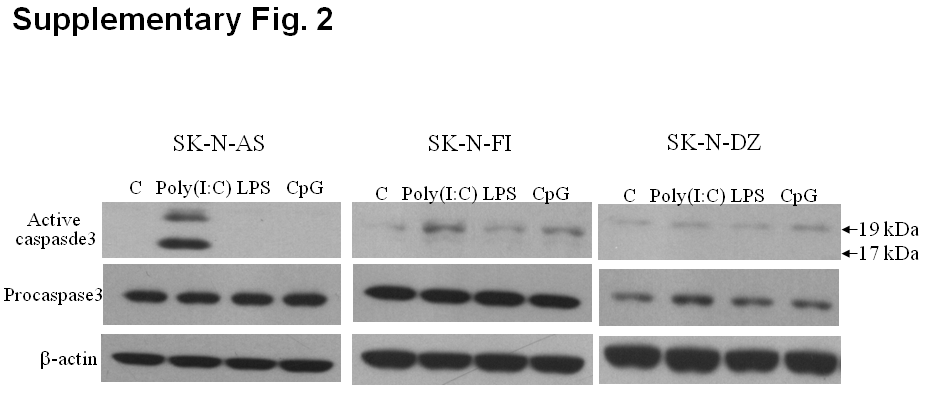

Supplement: Additional file 2 — Figure S2. Effect of TLR agonists on caspase-3 activation in NB cells. NB cells were treated with TLR agonists including TLR3 agonist (Poly(I:C); 50 μg/ml), TLR4 agonist (LPS; 10 ng/ml), and TLR9 agonist (CpG-ODN2006; 1 μM) for 24 h and subjected to immunoblot analysis of caspase-3 activation. [file 1423-0127-18-65-S2.TIFF]

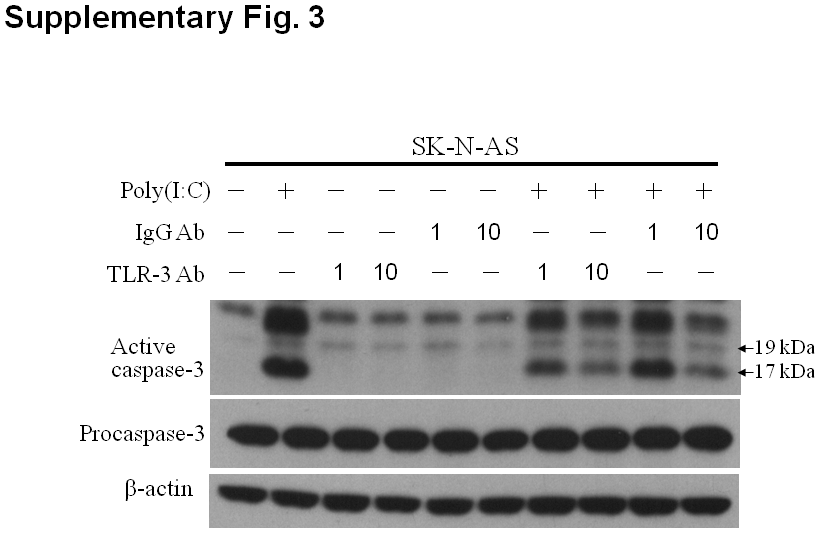

Supplement: Additional file 3 — Figure S3. Neutralization of TLR3 in SK-N-AS revealed that TLR3 specific antibody, in comparison with non-specific IgG control, down-regulated the expression of active caspase-3 expression. However, for uncertain reason, high dose of IgG may also exert some neutralization effect. [file 1423-0127-18-65-S3.TIFF]

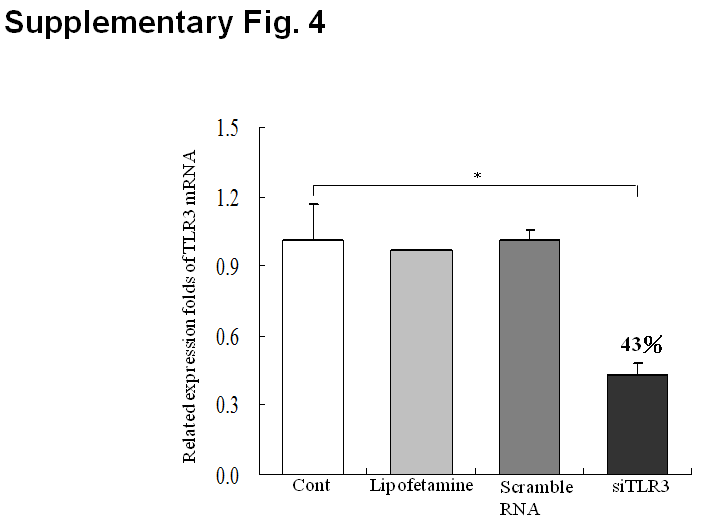

Supplement: Additional file 4 — Figure S4. Transfection of siRNA targeting TLR3 or scramble RNA was evaluated by using QRT-PCR to detect TLR3 mRNA. TLR3 mRNA expression decreased to 43% in SK-N-AS cells treated with siRNA targeting TLR3 (siTLR3), but not in scramble or lipofectamine control. [file 1423-0127-18-65-S4.TIFF]

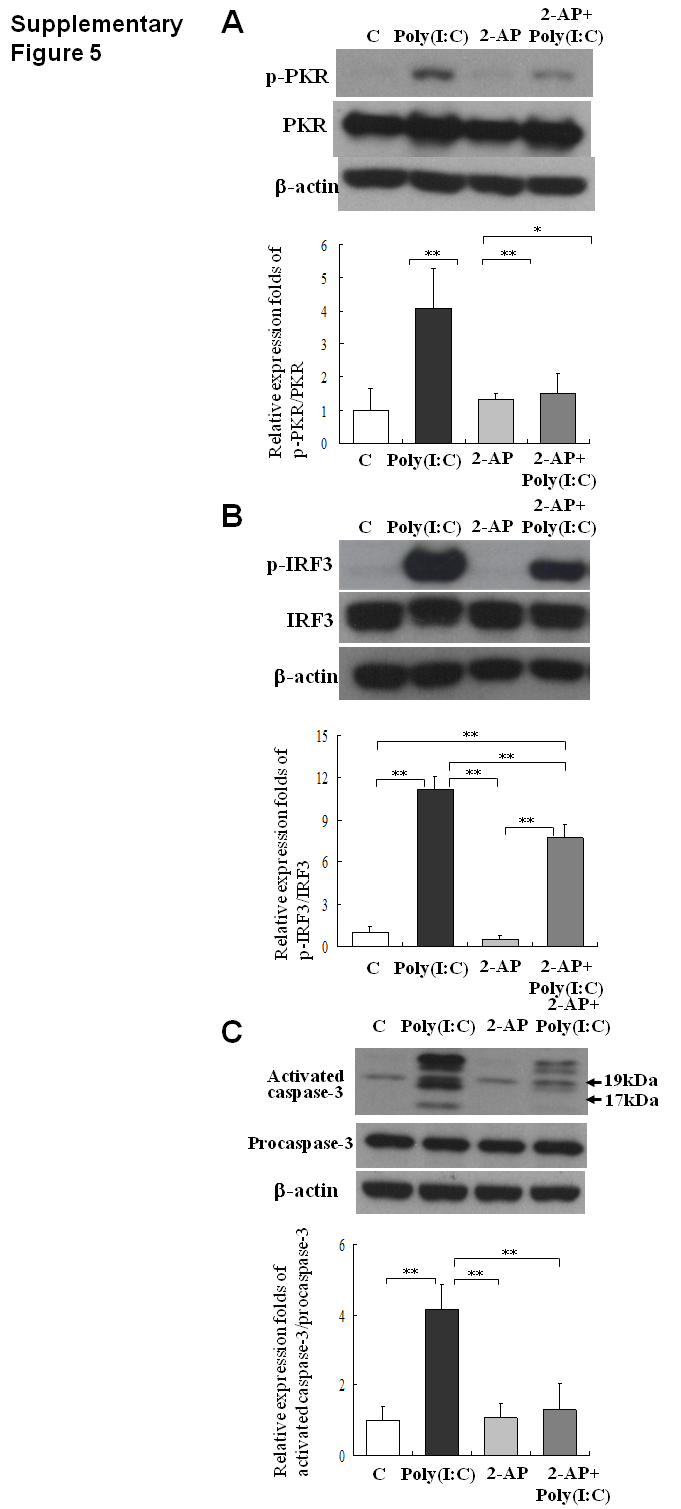

Supplement: Additional file 5 — Figure S5. Effect of PKR inhibitor, 2-aminopurine (2-AP), on poly(I:C)-induced suppression of PKR and IRF3 expression and caspase-3 activation in SK-N-AS cells. (A) NB cells were pretreated with 2-AP (10 mM) for 5 min, followed by treatment with 50 μg/ml poly(I:C) for 24 h, then harvested for immunoblot analysis. (A) Immunoblot analysis of total and phosphorylated PKR (top panel). Quantification of PKR activation was determined using phosphorylated PKR/total PKR (bottom panel). Data were mean ± SD from triplicate experiments. *: P < 0.05. **: P < 0.01. (B) Immunoblot analysis of total and phosphorylated IRF-3 (top panel). Quantification of IRF3 activation was determined using phosphorylated IRF3/total IRF3 (bottom panel). Data were mean ± SD from triplicate experiments. **: P < 0.01. (C) Immunoblot analysis of pro- and activated caspase-3 (top panel). Quantification of caspase 3 activation was determined using activated caspase-3 level over procaspase-3 level (bottom panel). Data were mean ± SD from triplicate experiments. **: P < 0.01. [file 1423-0127-18-65-S5.TIFF]
